# Front Line Applications and Future Directions of Immunotherapy in Small-Cell Lung Cancer

**DOI:** 10.3390/cancers13030506

**Published:** 2021-01-29

**Authors:** Selina K. Wong, Wade T. Iams

**Affiliations:** 1 Department of Medicine, Division of Hematology and Oncology, Nashville, TN 37232, USA; selina.wong@vumc.org; 2 Vanderbilt-Ingram Cancer Center, Vanderbilt University Medical Center, Nashville, TN 37232, USA

**Keywords:** small-cell lung cancer, immunotherapy, immune checkpoint inhibitors

## Abstract

**Simple Summary:**

Small-cell lung cancer (SCLC) is an aggressive malignancy with a high risk of recurrence and poor prognosis despite aggressive treatment. The use of immunotherapy has revolutionized the therapeutic landscape of SCLC with the introduction of novel, effective treatment options. Immune checkpoint inhibitors (ICIs) are the primary type of immunotherapy that have been used, first in the extensive-stage setting and now under investigation in the limited-stage setting. Here, we review the use of ICIs in SCLC as well as other emerging immunotherapy strategies.

**Abstract:**

After being stagnant for decades, there has finally been a paradigm shift in the treatment of small-cell lung cancer (SCLC) with the emergence and application of immune checkpoint inhibitors (ICIs). Multiple trials of first-line ICI-chemotherapy combinations have demonstrated survival benefit compared to chemotherapy alone in patients with extensive-stage SCLC, establishing this as the new standard of care. ICIs are now being applied in the potentially curative limited-stage setting, actively being investigated as concurrent treatment with chemoradiation and as adjuvant treatment following completion of chemoradiation. This review highlights the evidence behind the practice-changing addition of ICIs in the first-line setting of extensive-stage SCLC, the potentially practice-changing immunotherapy trials that are currently underway in the limited-stage setting, and alternate immunotherapeutic strategies being studied in the treatment of SCLC.

## 1. Introduction

Small-cell lung cancer (SCLC), which accounts for approximately 15% of lung cancers, is distinctive in its underlying biology, clinical course, and treatment approach. This aggressive malignancy exhibits rapid growth and early development of metastases, resulting in the majority of patients already having widespread, incurable disease at the time of presentation [1,2].

Early diagnosis and initiation of treatment is crucial in the management of SCLC. The Veteran’s Administration Lung Cancer Study Group categorizes SCLC into limited-stage and extensive-stage, with the differentiating feature being whether disease is limited to one hemithorax and therefore encompassable within a feasible radiation field. In less than 5% of cases, SCLC may be diagnosed in very early stages, wherein guidelines recommend definitive surgical resection followed by platinum-based adjuvant chemotherapy for stage I and selected stage IIA patients [2,3]. Otherwise, the mainstay of treatment for limited-stage SCLC (LS-SCLC) is concurrent chemoradiation (CRT) and prophylactic cranial irradiation (PCI). In extensive-stage SCLC (ES-SCLC), systemic therapies remain the cornerstone of treatment. SCLC carries a high risk of recurrence and poor prognosis despite aggressive treatment, with a median survival of 15–20 months for limited-stage and 8–13 months for extensive-stage disease [1,4]. 

Before the addition of immunotherapy to the treatment algorithm of SCLC, the therapeutic landscape of this disease had been stagnant for decades and lacked meaningful advances in treatment [5,6]. As of 2019, the combination of immune checkpoint inhibitor (ICI) and chemotherapy has been established as the standard of care in the first-line setting of ES-SCLC [7,8]. With the successful application of ICIs in the extensive-stage, this immunotherapeutic strategy is now being applied within the potentially curative, limited-stage setting. 

Immunotherapy with ICIs have transformed the therapeutic approach to SCLC and become a rapidly growing area of research. In this article, we review the rationale and data behind ICIs in ES-SCLC with a focus on the recent practice changing first line ICI use, and subsequently we detail the much-anticipated emerging data for ICIs in LS-SCLC. We also discuss the emerging studies evaluating novel immunologic strategies including chimeric antigen receptor (CAR) T cell therapy, vaccines, immunomodulators, and combination therapies. We will not explore the data for the use of ICIs in patients with relapsed SCLC in this review, rather we refer readers to previous reviews on the topic [9,10,11] and note that the directionality of the field of ICIs in SCLC is moving towards an emphasis on the front line setting. Of note, randomized trials have failed to demonstrate an overall survival (OS) benefit for ICIs compared to chemotherapy for relapsed SCLC [12,13] and the relapsed SCLC indication for nivolumab has been recently withdrawn (though this remains a category 3 treatment option in National Comprehensive Cancer Network guidelines) [14,15].

## 2. Rationale for Immunotherapy in Small-Cell Lung Cancer

The immune system plays a critical role in cancer pathogenesis. Normally, there exists a fine balance between the ability to recognize “self” to avoid autoimmunity and recognizing “non-self” to appropriately mount a response against foreign entities. By taking advantage of mechanisms utilized by the immune system to supress autoimmunity, cancer cells manage to establish sufficient immune tolerance so as to evade antitumor responses that would normally be activated against them [16,17]. 

High tumor mutational load is thought to facilitate the activation of the adaptive immune system through the production and subsequent presentation of tumor-specific neoantigens to T cells [18,19,20]. Although SCLC is characterized by high somatic mutational load [21,22], in part due to its strong association with tobacco exposure [23], there is accumulating evidence that SCLC exerts immunosuppressive effects. T cells, specifically effector T cells (Teffs) and regulatory T cells (Tregs), are key players in mediating the antigen-specific immune response pathway. Whereas the activation of Teffs leads to antitumor activity, Tregs act to downregulate immune responses as a means of preventing autoimmunity [24]. Abnormally high levels of Tregs relative to Teffs have been observed in the setting of cancer, hypothesized to be a means by which antitumor responses become downregulated [25,26]. Koyama et al. analyzed 35 peripheral blood samples of patients with SCLC, finding the Teffs to Tregs ratio to be prognostic. Not only did they observe significantly more Teffs in LS-SCLC and conversely more Tregs in ES-SCLC, but also that long-term survivors of SCLC maintained a high Teffs to Tregs ratio. On the other hand, this ratio was low among patients with recurrent disease [27]. 

Similarly, immune cell infiltration in the tumor microenvironment has demonstrated prognostic value in SCLC, with higher levels of T cells, CD8 cells, and CD45-positive T cells being detected in long-term survivors [28,29,30]. Most recently was a case-control study by Muppa et al., comparing resected tumors of 23 long-term SCLC survivors (>4 years) and 18 survivors with expected survival time of less than 2 years. Both the absolute number of tumor-infiltrating lymphocytes (TILs) and the ratio of these relative to immunosuppresive immune cells were found to be different in the two cohorts. Not only did long-term survivors have significantly more TILs, they notably also had higher numbers of suppressive cells (including monocytes, lymphocytes, and macrophages) albeit lower ratios of CD68-positive macrophages to CD3-positive T lymphocytes compared to those with less than 2 year survival [29]. Potential mechanisms by which SCLC is able to evade the immune system include decreased levels of TILs and loss of expression of major histocompatibility complex (MHC) class II [31,32]. 

The involvement of the immune system in the pathophysiology of SCLC is also evident in the association between occurrence of paraneoplastic syndromes (PNS), such as Lambert-Eaton myasthenic syndrome (LEMS), and long-term prognosis. Patients with SCLC who develop LEMS have a more favorable prognosis than those who do not develop the neurological illness [33]. A recent retrospective review of 145 SCLC patients demonstrated that those who developed a neurologic PNS were found to have both increased TILs and improved median overall survival (24 vs. 12 months) compared to those without PNS [34]. These accumulating data provide the basis for the application of immunotherapeutic strategies in SCLC.

## 3. Immune Checkpoint Inhibitors

The most extensively utilized type of immunotherapy has been ICIs, specifically those targeting the pathways involving programmed death-1 (PD-1) and cytotoxic T-lymphocyte associated protein 4 (CTLA-4). By expressing ligands that bind PD-1 or CTLA-4 receptors, tumors take advantage of the resulting negative co-stimulatory signals that inhibit T-cell activation and prevent downstream cell-mediated destruction. ICIs reinstitute appropriate antitumor response by inhibiting the binding of these receptors [35,36]

## 4. Extensive-Stage Small-Cell Lung Cancer 

The benefit of combination ICI with chemotherapy in the first-line setting of ES-SCLC is well established and has been studied in four phase III trials (IMpower133, CASPIAN, KEYNOTE-604, and CA184-156) and more recently a phase II ECOG-ACRIN EA5161 trial (Table 1).

In the phase III, placebo-controlled, randomized IMpower133 trial, the anti-programmed death ligand 1 (PD-L1) agent atezolizumab or placebo was combined with the chemotherapy backbone of carboplatin and etoposide for four cycles, followed by either atezolizumab or placebo maintenance [7]. Testing for PD-L1 expression was not required nor were patients stratified, but exploratory analyses did include assessment of efficacy in relation to blood-based tumor mutational burden. A total of 403 previously untreated patients with ES-SCLC were enrolled, of which 201 patients received atezolizumab plus chemotherapy. Although PCI was permitted during the maintenance phase, consolidative thoracic radiotherapy was not. The two primary endpoints of the trial, progression-free survival (PFS) and OS, demonstrated statistical significance in favor of the atezolizumab plus chemotherapy cohort: PFS 5.2 months vs. 4.3 months (HR 0.77, 95% confidence interval [CI] 0.62–0.96, *p* = 0.02) and OS 12.3 months vs. 10.3 months (HR 0.70, 95% CI 0.54–0.91, *p* = 0.007). Incidence of grade 3 or 4 adverse events was balanced between the atezolizumab-containing (56.6%) and placebo (56.1%) arms. Though OS was already statistically significant at interim analysis, an updated exploratory OS analysis was presented at the 2019 European Society of Medical Oncology Congress. At a median follow-up of 22.9 months, improvement seen in the atezolizumab-containing arm persisted with 18-month OS of 24% vs. 21% [37]. 

The efficacy of combining durvalumab with or without tremelimumab with chemotherapy was investigated in the phase III trial CASPIAN [8]. Patients were randomized to receive durvalumab plus platinum-etoposide, durvalumab, tremelimumab (a CTLA-4 inhibitor) plus platinum-etoposide, or platinum-etoposide alone, followed by maintenance. PCI was permitted at the physician’s discretion in the chemotherapy alone group only. At interim analysis in 2019, results were presented for the durvalumab plus platinum-etoposide vs. the platinum-etoposide only groups. The primary endpoint of OS had been met and was in favor of the durvalumab group: 13.0 months vs. 10.3 months, HR 0.73, 95% CI 0.59–0.91, *p* = 0.0047. PFS, a secondary endpoint, was 5.1 months in the durvalumab arm vs. 5.4 months in the chemotherapy only arm, HR 0.78, 95% CI 0.65–0.94. After a median follow up of 25.1 months, the updated efficacy analyses of durvalumab plus platinum-etoposide as well as the initial results of the tremelimumab-containing cohort were presented at the 2020 American Society of Clinical Oncology meeting [38]. The durvalumab-containing arm continued to demonstrate superior OS compared to platinum-etoposide alone, 12.9 months vs. 10.5 months, HR 0.75, 95% CI 0.62–0.91, *p* = 0.0032 and this benefit was seen regardless of whether carboplatin or cisplatin was used. On the other hand, the tremelimumab arm did not reach statistical significance with median OS of 10.4 months vs. 10.5 months with chemotherapy alone (HR 0.82, 95% CI 0.68–1.00, *p* = 0.0451). Two-year survival rates were 22.2% in the durvalumab arm, 23.4% in the tremelimumab arm, and 14.4% with platinum-etoposide alone. Occurrence of grade 3 or 4 adverse events was slightly higher with the dual ICI plus chemotherapy arm (70.3%), but was otherwise well balanced between the durvalumab and chemotherapy alone arms (62.3% and 62.8%, respectively).

At the 2020 American Society of Clinical Oncology meeting, the efficacy of nivolumab given in combination with platinum-etoposide vs. platinum-etoposide alone for first-line treatment of ES-SCLC was presented from the randomized phase II ECOG-ACRIN EA5161 trial [39]. The addition of nivolumab to chemotherapy, followed by nivolumab maintenance, improved both PFS (5.5 months vs. 4.6 months, HR 0.65, 95% CI 0.46–0.91, *p* = 0.012) and OS (11.3 months vs. 8.5 months, HR 0.67, 95% CI 0.46–0.98, *p* = 0.038). No new safety signals were observed, with the incidence of grade 3 or 4 adverse events 77% with nivolumab vs. 62%.

KEYNOTE-604 investigated the addition of pembrolizumab, an anti-PD-1 agent, to platinum-etoposide as first-line treatment of ES-SCLC [40]. Eligible patients received chemotherapy plus either pembrolizumab or placebo, followed by maintenance. Although the addition of pembrolizumab improved PFS (4.5 months vs. 4.3 months, HR 0.75, 95% CI 0.61–0.91, *p* = 0.0023), a statistically significant difference in OS was narrowly missed (10.8 months vs. 9.7 months, HR 0.80, 95% CI 0.64–0.98, *p* = 0.0164). Pembrolizumab was generally well tolerated, with a grade 3 or 4 adverse event rate of 76.7% with pembrolizumab vs. 74.9% with chemotherapy alone.

After promising results from a phase II trial wherein ipilimumab was administered in a phased approach after initial exposure to chemotherapy [41], Reck et al. undertook the multicenter, randomized, double-blinded phase III CA184-156 trial to explore the utility of adding an anti-CTLA-4 agent ipilimumab to platinum-etoposide, followed by ipilimumab or placebo maintenance [42]. During the induction phase (6 cycles), patients in both arms received platinum-etoposide throughout cycles 1 to 4. In cycles 3 and 4, patients were randomized to receive either the addition of ipilimumab or placebo. Patients then received only ipilimumab or placebo in the final cycles 5 and 6 of induction, followed by maintenance among patients who achieved either a complete or partial response. This study failed to meet its primary endpoint of OS: 11.0 months in the ipilimumab arm vs. 10.9 months in the placebo arm, HR 0.94, 95% CI 0.81–1.09, *p* = 0.3775.

## 5. Limited-Stage Small-Cell Lung Cancer

Having demonstrated benefit in the extensive-stage setting, ICIs are now under rigorous investigation in the limited-stage setting. Even after curative-intent treatment, there is a high recurrence risk of approximately 70% at 5 years [43]. Being that the primary treatment modality for LS-SCLC is concurrent CRT [44,45], the addition of ICIs is being investigated both in the concurrent setting with CRT and in the adjuvant setting after definitive CRT (Table 2).

In the concurrent setting, data from a single-center, open label, phase I/II trial of pembrolizumab given with concurrent chemoradiation in the treatment of LS-SCLC and other neuroendocrine tumors was recently published [46]. The primary endpoint was safety (dose-limiting toxicities) and secondary endpoints included PFS, OS, and tumor response. Pembrolizumab was started concurrently with CRT (45Gy radiotherapy and platinum-etoposide chemotherapy) and continued for up to 16 cycles. PCI was permitted at the physician’s discretion, with a total of 27 (61%) patients who underwent PCI. A total of 40 patients were treated with at least one cycle of pembrolizumab: median PFS was 19.7 months (95% CI 8.8–30.5) and OS was 39.5 months (95% CI 8.0–71.0). At the median follow-up time of 23.1 months, 20 (50%) patients had developed disease progression. Thirty-three of the 40 patients were evaluable for response and had an ORR of 79%. Three patients experienced grade 4 toxicities (2 neutropenia and 1 respiratory failure) while the most common grade 3 toxicities were neutropenia (5 patients) and anemia (5 patients). A pneumonitis rate of 15% was seen (three grade 2 and three grade 3). The authors concluded that this regimen not only yielded favorable outcomes, but was also well tolerated. The safety of combination immunotherapy and radiation in this trial was comparable to that of the CONVERT trial of once-daily vs. twice-daily chemoradiation in LS-SCLC: pneumonitis 15% vs. 21% in CONVERT and esophagitis 42.5% vs. 81% in CONVERT [43]. Given these results from the first prospective trial of concurrent ICI and CRT in LS-SCLC, the results of an ongoing phase II/III trial LU005 (NCT03811002) of atezolizumab plus CRT vs. CRT alone in LS-SCLC are much anticipated.

In the adjuvant setting where ICIs are given as maintenance following curative CRT, multiple trials are underway including the phase II ACHILES (NCT03540420), phase III ADRIATIC (NCT03703297), and phase II STIMULI (NCT02046733) trials. Respectively, these trials are investigating the efficacy of maintenance atezolizumab, durvalumab and/or tremelimumab, and nivolumab plus ipilimumab following completion of chemoradiation (Table 2). Given the practice-changing findings from the PACIFIC trial of maintenance durvalumab after definitive CRT in unresectable stage III non-small cell lung cancer (NSCLC) [47,48], the results of these SCLC trials are eagerly awaited.

## 6. Predictive Biomarkers 

Unlike in NSCLC where PD-L1 expression is used to guide first-line treatment with ICI monotherapy [49], an equivalent predictive biomarker to inform the use of ICIs in SCLC remains elusive. PD-L1 expression is evaluated by immunohistochemistry, with multiple clinical trial validated assays that have been approved as companion diagnostics. There are inherent limitations of this biomarker [50], including its heterogeneity both temporally and spatially (intratumoral and intertumoral), inter-assay variability, and potential discordance between surgical resected and matched biopsy specimens [51,52,53]. Furthermore, PD-L1 expression may vary depending on whether expression is determined on tumor cells, immune cells, and/or stroma.

In the first-line setting, IMpower133, CASPIAN, and KEYNOTE-604 did not demonstrate a strong association between PD-L1 expression and ICI efficacy. IMpower133 included an exploratory analysis of PD-L1 expression (on immune or tumor cells) and survival. The PD-L1 evaluable population comprised 34% (137/403 patients) of the intention-to-treat population, with efficacy analyses conducted using PD-L1 cut-offs of 1% and 5%. An OS benefit favoring the addition of atezolizumab to chemotherapy was seen in the PD-L1 < 1% cohort (10.2 months vs. 8.3 months), but not in the PD-L1 ≥ 1% and ≥5% cohorts [37,51,52,53]. In the CASPIAN trial, only 5% of patients had PD-L1 expression ≥1% in tumor cells and 22% of patients with PD-L1 expression ≥1% in immune cells. The investigators evaluated PD-L1 expression as a continuous variable and did not observe any impact on ORR, PFS, or OS between treatment arms [54]. KEYNOTE-604 measured PD-L1 expression using the combined positive score (CPS), which takes into account both tumors cells and tumor-infiltrating cells. Similarly, no differences in PFS or OS were observed based on PD-L1 expression [40].

Some evidence has suggested that the expression of PD-L1 in the tumor microenvironment, specifically on host cells as opposed to tumors cells, may in fact be a better predictor of response to ICIs [55,56]. Schultheis et al. examined 94 cases of SCLC, among which none of them demonstrated PD-L1 expression on tumor cells but 17 (18%) of them expressed PD-L1 within the stroma [57]. The importance of the PD-1/PD-L1 pathway in the tumor microenvironment was also demonstrated in a study of 193 patients with large cell neuroendocrine carcinoma or SCLC examined for PD-L1 expression on tumor cells and tumor-infiltrating immune cells. No correlation was seen between PD-L1 expression on tumor cells and that on immune cells. Patients with PD-L1 expression on immune cells had significantly longer PFS than those without (11.3 months vs. 7.0 months, *p* = 0.02) and notably, this correlation with survival was not demonstrated with PD-L1 expression on tumor cells [58]. 

Tumor mutational burden (TMB), a measure of non-synonymous somatic mutations, has been shown across multiple cancer types to be associated with improved OS after treatment with ICIs [59]. Its utility in guiding the treatment of SCLC has proven to be inconclusive, similar to PD-L1 expression. High TMB is thought to result in a higher neoantigen load which allows for T cell activation and downstream antitumor effects [60]. Ricciuti et al. collected data from 52 patients with relapsed or refractory SCLC who went on to receive treatment with an ICI. Patients with high TMB (above 50th percentile) achieved significantly longer PFS and OS compared to their low TMB (below 50th percentile) counterparts: median PFS 3.3 months vs. 1.2 months and OS 10.4 months vs. 2.5 months [61]. Based on findings by Gandara et al. showing the ability of blood-based TMB (bTMB) to identify patients who derive clinically significant improvements in PFS from atezolizumab in second-line or later advanced NSCLC [62], the utility of bTMB as a predictive biomarker was an exploratory analysis in IMpower133. Blood-based TMB analyses with cut-off values of 10 and 16 mutations per megabase (Mb) were possible in 351 of the 403 patients (93.8%). Consistent OS and PFS benefits were demonstrated across all subgroups in favor of atezolizumab plus chemotherapy, although the <10 mutations/Mb and ≥16 mutations/Mb subgroups did not reach statistical significance [7]. 

## 7. Other Immunotherapeutic Approaches 

Apart from ICIs, there are a variety of different mechanisms by which the immune system can be harnessed to mount an antitumor response. These include CAR T cell therapy, bispecific T cell engagers (BiTEs), antibody-drug conjugates, and immunomodulators (Table 3).

While endogenous T cell activation is dependent on antigen presentation by MHC class I, T cell-based therapy is an MHC-independent therapeutic strategy. Chimeric antigen receptors are recombinant receptors for tumor-specific antigens, which then become engineered into T cells to enable expression, expansion, and anti-tumor specificity [63]. Multiple cell surface molecules have emerged as potential therapeutic targets, including CD56 [64] and CD47 [65], both of which are highly expressed on the surface of SCLC cells. Similarly, delta-like ligand 3 (DLL3) is an inhibitory Notch pathway ligand that is upregulated and overexpressed in high-grade neuroendocrine tumors [66]. While it is expressed in over 80% of SCLC, there is little to no expression on normal lung tissue, therefore making it an attractive therapeutic target [67,68]. DLL3-targeted CAR T cell-based therapy, AMG 119, is being studied in an ongoing phase I trial of patients with relapsed/refractory SCLC (NCT03392064). Another DLL-3 targeted immunotherapy that has been developed is the BiTE, AMG 757. BiTEs are recombinant bispecific proteins that simultaneously target a T-cell surface molecule (such as CD3) and a tumor-specific surface antigen, thereby facilitating T cell adherence and antitumor response independent of MHC [69]. AMG 757 alone and in combination with pembrolizumab is currently being evaluated in a phase I trial (NCT03319940). Rovalpituzumab tesirine (Rova-T), a DLL3-targeted antibody-drug conjugate [70], has failed to establish a role in the treatment of SCLC after limited activity was demonstrated in the third-line (phase II single-arm TRINITY), second-line (phase III TAHOE), and first-line maintenance following platinum-based chemotherapy (phase III MERU) [71,72].

Vaccines are a potentially promising strategy in the management of SCLC and remain under investigation. Vaccines are designed with the intent of exposing host cells to tumor antigens, thereby potentiating an adaptive immune response. Multiple vaccines have been studied thus far, including fucosyl GM-1, GD3 ganglioside, polysialic acid, and dendritic cell-based p53 [9,73,74].

The utility of immunomodulatory agents such as interleukin-2 and interferon has been studied and failed to demonstrate benefit [74]. Most recently, the efficacy of lefitolimod as maintenance therapy after first-line chemotherapy was investigated in phase II trial IMPULSE. The mechanism of action for lefitolimod, a toll-like receptor (TLR) 9 agonist, is the activation of innate immunity via stimulation of cytokine production [75]. IMPULSE failed to demonstrate an OS benefit in the intention-to-treat population, although a subgroup analysis of patients with a low frequency of activated CD86+ B cell revealed an OS benefit signal, HR 0.53, 95% CI 0.26–1.08 [76].

Multiple novel immunotherapeutic strategies are emerging to investigate combination approaches with ICIs (Table 3). TIM-3 and LAG-3 are two immune checkpoint molecules that contribute to immune tolerance. Their upregulation has been observed and implicated in the development of resistance to PD-1 blockade [77,78]. Anti-TIM-3 agents are being investigated as monotherapy, in combination with anti-PD-1/anti-CTLA-4 agents, and dual blockade using bispecific antibodies [79]. A phase II trial of anti-PD-1 agent, spartalizumab, and anti-LAG-3 agent, LAG525, reported preliminary efficacy analyses across seven tumor types including SCLC. Promising activity in SCLC was reported, although final results have yet to be presented [80]. T cell immunoreceptor with immunoglobulin and ITIM domains (TIGIT) and its ligands CD155 and CD112, is another immune checkpoint pathway being targeted for anticancer therapy [81]. In ES-SCLC, the SKYSCRAPER-02 (NCT04256421) is a phase III, randomized, double-blind, placebo-controlled trial underway to investigate the addition of an anti-TIGIT agent, tiragolumab, to first-line atezolizumab, carboplatin, plus etoposide.

Other immunomodulatory agents have also been combined with ICIs, for example N-803 and BNT411. N-803, an interleukin-15 superagonist, was studied in combination with nivolumab in metastatic NSCLC [82], and is currently being investigated in combination with PD-1/PD-L1 agents in the setting of advanced solid tumors (including SCLC) that have progressed on or after single-agent checkpoint inhibitor in the QUILT-3.055 trial (NCT03228667). The safety and efficacy of BNT411, a TLR7 agonist, is being explored as monotherapy and in combination with atezolizumab, carboplatin, and etoposide in ES-SCLC (NCT04101357).

The small molecules cyclin-dependent kinases (CDK) 4/6 inhibitors and poly (ADP-ribose) polymerase (PARP) inhibitors have both been applied in combination with an ICI in the treatment of SCLC. The efficacy of trilaciclib, a CDK4/6 inhibitor, administered with first-line atezolizumab, carboplatin, and etoposide in ES-SCLC is being investigated in NCT03041311. Durvalumab in combination with PARP inhibitor, olaparib, was studied in a single-arm phase II study of relapsed SCLC and ultimately did not meet the pre-set bar for efficacy [83], but this strategy of simultaneous ICI and PARP inhibition continues to be explored [84].

Although platinum and etoposide are the most established chemotherapies in the treatment of SCLC, lurbinectedin is an alkylating agent that is being investigated in combination with ICIs including pembrolizumab (NCT04358237), nivolumab and ipilimumab (NCT04610658), and atezolizumab (NCT04253145). 

Mechanistically, vaccines may potentiate the effects of ICIs given they both act on the adaptive immune system. Ongoing trials include nivolumab plus ipilimumab with a dendritic p53 vaccine (NCT03406715) and atezolizumab in combination with a dendritic cell vaccine (NCT04487756). 

Finally, another novel approach to the treatment of SCLC that has shown evidence of antitumor effect in a phase I trial is combination of lutetium-labeled somatostatin analog in combination with nivolumab [85]. This approach takes advantage of somatostatin receptors that are expressed by some neuroendocrine tumors, including SCLC. As we further our understanding of resistance mechanisms to PD-1/CTLA-4 agents and develop novel therapies, additional combination studies are likely to emerge. Understanding optimal sequencing of treatments and how to take advantage of additive or synergistic effects of drugs will be crucial.

## 8. Conclusions

The treatment landscape of SCLC has been revolutionized by the integration of immunotherapy. While ICIs have established a clear role in the front line treatment of ES-SCLC, the results of several ongoing trials investigating their efficacy in the curative, limited-stage setting are much anticipated. Ultimately despite the substantial advances made with immunotherapies, the reality of SCLC remains that the majority of patients will eventually relapse and experience a poor prognosis. Continued drug development of novel targeted therapies will be crucial, both for use in combination with immunotherapy and/or as later line therapy in the setting of immunotherapy refractory disease.

## Figures and Tables

**Table 1:** Summary of first-line ICI trials in ES-SCLC.

Trial	Phase	No. of Patients	Treatment	FDA Approval	Primary Endpoint(s) (Met?)	PFS	OS	ORR (%)	Grade 3/4 Adverse Events (%)
IMpower133 (Horn et al.)	III	403	Atezolizumab + carboplatin/etoposide vs. carboplatin/etoposide	Yes	OS (yes) PFS (yes)	5.2 vs. 4.3 months (HR 0.77, 95%CI 0.62–0.96, *p* = 0.02)	12.3 vs. 10.3 months (HR 0.70, 95%CI 0.54–0.91, *p* = 0.007)	60.2 vs. 64.4	56.6 vs. 56.1
CASPIAN * (Paz-Ares et al.)	III	805	Durvalumab + platinum/etoposide vs. platinum/etoposide	Yes	OS (yes)	5.1 vs. 5.4 months (HR 0.78, 95% CI 0.65–0.94)	13.0 vs. 10.3 months (HR 0.73, 95%CI 0.59–0.91, *p* = 0.0047)	79 vs. 70	62 vs. 62
ECOG-ACRIN EA5161 (Leal et al.)	II	160	Nivolumab + platinum/etoposide vs. platinum/etoposide	No	PFS (yes)	5.5 vs. 4.6 months (HR 0.65, 95%CI 0.46–0.91, *p* = 0.012)	11.3 vs. 8.5 months (HR 0.67, 95%CI 0.46–0.98, *p* = 0.038)	52.3 vs. 47.7	77 vs. 62
KEYNOTE-604 (Rudin et al.)	III	453	Pembrolizumab + platinum/etoposide vs. platinum/etoposide	No	OS (no) PFS (yes)	4.5 vs. 4.3 months (HR 0.75, 95% CI 0.61–0.91, *p* = 0.0023)	10.8 vs. 9.7 months (HR 0.80, 95%CI 0.64–0.98, *p* = 0.0164)	70.6 vs. 61.8	76.7 vs. 74.9
CA184-156 (Reck et al.)	III	1132	Ipilimumab + platinum/etoposide vs. platinum/etoposide	No	OS (no)	4.6 vs. 4.4 months (HR 0.85, 95%CI 0.75–0.97, *p* = 0.0161)	11.0 vs. 10.9 months (HR 0.94, 95%CI 0.81–1.09, *p* = 0.3775)	62 vs. 62	48 vs. 45

* treatment arm containing durvalumab plus tremelimumab plus platinum plus etoposide did not reach statistical significance.

**Table 2:** Summary of ICI trials in LS-SCLC.

Trial	Phase	Status	Setting	Treatment	Primary Endpoint(s)	Target Enrolment	Start Date–Estimated Completion Date
Welsh et al. 2020	I/II	Complete	Concurrent with CRT	Pembrolizumab + concurrent CRT	Safety: no grade 5 toxicity, pneumonitis 15%, esophagitis 42.5% * PFS was 19.7 months (95% CI 8.8–30.5) * OS was 39.5 months (95% CI 8.0–71.0) * ORR of 79%	40	Completed
LU-005 (NCT03811002)	II/III	Ongoing	Concurrent with CRT	Atezolizumab + concurrent CRT vs. CRT	OS, PFS	506	28 May 2019–28 December 2026
ACHILES (NCT03540420)	II	Ongoing	Maintenance after CRT	Atezolizumab vs. observation	2-year survival	212	31 July 2018–December 2026
ADRIATIC (NCT03703297)	III	Ongoing	Maintenance after CRT	Durvalumab vs. durvalumab + tremelimumab vs. placebo	OS, PFS	724	27 September 2018–10 May 2024
STIMULI (NCT02046733)	II	Ongoing	Maintenance after CRT	Nivolumab + ipilimumab vs. observation	OS, PFS	174	28 July 2014–January 2022

* Secondary outcomes.

**Table 3:** Overview of other immunotherapeutic approaches, used alone or in combination with ICIs, in the treatment of SCLC.

Type	Examples
CAR T cell therapy	AMG 119 (targeting DLL-3)
Bispecific T cell engager	AMG 757 (targeting DLL-3)
Antibody-drug conjugate	Rovalpituzumab tesirine (targeting DLL-3)
Immunomodulators	Interleukin-2 Interferon Lefitolimod (TLR9 agonist) N-803 (interleukin-15 agonist) BNT411 (TLR7 agonist)
Vaccine	Fucosyl GM-1 GD3 ganglioside Polysialic acid Dendritic cell-based p53
Immune checkpoint	TIM-3 LAG-3 TIGIT
Small molecule	CDK4/6 inhibitor PARP inhibitor
Alkylating agent	Lurbinectedin
Other	Lutetium-labeled somatostatin analog

## Data Availability

No new data were created or analyzed in this study. Data sharing is not applicable to this article.
